# Intravenous methylprednisolone pulse as a treatment for hospitalised severe COVID-19 patients: results from a randomised controlled clinical trial

**DOI:** 10.1183/13993003.02808-2020

**Published:** 2020-12-24

**Authors:** Maryam Edalatifard, Maryam Akhtari, Mohammadreza Salehi, Zohre Naderi, Ahmadreza Jamshidi, Shayan Mostafaei, Seyed Reza Najafizadeh, Elham Farhadi, Nooshin Jalili, Masoud Esfahani, Besharat Rahimi, Hossein Kazemzadeh, Maedeh Mahmoodi Aliabadi, Tooba Ghazanfari, Mohammadreza Sattarian, Hourvash Ebrahimi Louyeh, Seyed Reza Raeeskarami, Saeidreza Jamalimoghadamsiahkali, Nasim Khajavirad, Mahdi Mahmoudi, Abdolrahman Rostamian

**Affiliations:** 1 Advanced Thoracic Research Center, Tehran University of Medical Sciences, Tehran, Iran; 2 Rheumatology Research Center, Tehran University of Medical Sciences, Tehran, Iran; 3 Inflammation Research Center, Tehran University of Medical Sciences, Tehran, Iran; 4 Dept of Infectious and Tropical Medicines, Tehran University of Medical Sciences, Tehran, Iran; 5 Dept of Internal Medicine, Isfahan University of Medical Sciences, Isfahan, Iran; 6 Dept of Biostatistics, School of Health, Kermanshah University of Medical Sciences, Kermanshah, Iran; 7 Rheumatology Research Center, Imam Khomeini Hospital, Tehran University of Medical Sciences, Tehran, Iran; 8 Dept of Internal Medicine, School of Medicine, Zanjan University of Medical Sciences, Zanjan, Iran; 9 Dept of Clinical Pharmacy, Faculty of Pharmacy, Tehran University of Medical Sciences, Tehran, Iran; 10 Dept of Laboratory, Imam Khomeini Hospital Complex, Tehran University of Medical Sciences, Tehran, Iran; 11 Immunoregulation Research Center, Shahed University, Tehran, Iran; 12 Simorgh Clinical Laboratory, Tehran, Iran; 13 Dept of Rheumatology, Imam Khomeini Hospital Complex, Tehran University of Medical Sciences, Tehran, Iran; 14 Dept of Pediatrics, Tehran University of Medical Sciences, Tehran, Iran; 15 Ziaeian Hospital, Tehran University of Medical Sciences, Tehran, Iran; 16 Dept of Internal Medicine, School of Medicine, Tehran University of Medical Sciences, Tehran, Iran; 17 These two authors contributed equally as first authors; 18 These three authors contributed equally as lead authors and supervised the work

## Abstract

**Introduction:**

There are no determined treatment agents for severe COVID-19. It is suggested that methylprednisolone, as an immunosuppressive treatment, can reduce the inflammation of the respiratory system in COVID-19 patients.

**Methods:**

We conducted a single-blind, randomised controlled clinical trial involving severe hospitalised patients with confirmed COVID-19 at the early pulmonary phase of the illness in Iran. The patients were randomly allocated in a 1:1 ratio by the block randomisation method to receive standard care with methylprednisolone pulse (intravenous injection, 250 mg·day^−1^ for 3 days) or standard care alone. The study end-point was the time of clinical improvement or death, whichever came first. Primary and safety analysis was done in the intention-to-treat (ITT) population.

**Results:**

68 eligible patients underwent randomisation (34 patients in each group) from April 20, 2020 to June 20, 2020. In the standard care group, six patients received corticosteroids by the attending physician before the treatment and were excluded from the overall analysis. The percentage of improved patients was higher in the methylprednisolone group than in the standard care group (94.1% *versus* 57.1%) and the mortality rate was significantly lower in the methylprednisolone group (5.9% *versus* 42.9%; p<0.001). We demonstrated that patients in the methylprednisolone group had a significantly increased survival time compared with patients in the standard care group (log-rank test: p<0.001; hazard ratio 0.293, 95% CI 0.154–0.556). Two patients (5.8%) in the methylprednisolone group and two patients (7.1%) in the standard care group showed severe adverse events between initiation of treatment and the end of the study.

**Conclusions:**

Our results suggest that methylprednisolone pulse could be an efficient therapeutic agent for hospitalised severe COVID-19 patients at the pulmonary phase.

## Abstract 2

**This study showed that methylprednisolone pulse administration at the beginning of the early pulmonary phase of illness decreased the mortality rate and improved pulmonary involvement, oxygen saturation and inflammatory markers in COVID-19 patients** https://bit.ly/3hik4JB

## Introduction

The world is experiencing the pandemic of a novel coronavirus-induced respiratory illness named COVID-19. The disease is caused by severe acute respiratory syndrome coronavirus 2 (SARS-CoV-2), which belongs to the genus Betacoronavirus [1]. Betacoronaviruses are positive single-stranded RNA viruses that have caused two other severe outbreaks, *i.e.* Middle East respiratory syndrome (MERS) and severe acute respiratory syndrome (SARS), over recent decades [2]. COVID-19 has rapidly spread across the world and the number of infected people is increasing since it was first discovered in China in late 2019. The virus can be transmitted from person to person during the 2–14-day asymptomatic incubation period. The signs of illness range from fever, dry cough, fatigue, myalgia and mild respiratory tract symptoms to serve manifestations, including shortness of breath, pneumonia and acute respiratory distress syndrome (ARDS), dependent on the patient's age, genetics factors and the function of the immune system [3, 4]. Extrapulmonary involvements (*e.g.* hepatic and gastrointestinal) are also present in some patients [5].

Typically, in the early phase of the disease, specific and proper immune system responses eliminate virus reproduction and prevent disease progression to the hyperinflammation phase. If the infection is not eliminated by the appropriate and strong immune responses, the disease enters the severe inflammatory response phase when a cytokine storm and elevated inflammatory markers produced by innate immune cells induce pulmonary fibrosis, shortness of breath, reduction in oxygen saturation and systemic injuries, resulting in ARDS and the patient's death [6]. Cytokine storm induction by SARS-CoV-2 was confirmed in COVID-19 patients in the intensive care unit (ICU), and elevated plasma levels of inflammatory cytokines have been associated with disease severity and prognosis [7, 8].

ARDS is the main reason for death in COVID-19 patients and there are no efficient specific treatment agents for the disease [8]. It is suggested that glucocorticoids and immunosuppressive treatment can reduce the inflammation of the respiratory system and prevent the cytokine storm and ARDS induction in COVID-19 patients [9]. Methylprednisolone is a glucocorticoid medication used to suppress autoimmune and inflammatory responses in rheumatic diseases [10]. Previously, methylprednisolone was administrated in SARS and MERS patients, and the results were controversial [11–13]; however, glucocorticoid administration in COVID-19 patients in the hyperinflammation stage is likely to have survival benefits due to cytokine storm suppression. Hence, in this study, we investigated the methylprednisolone pulse effect as a glucocorticoid therapy on the treatment, clinical symptoms and laboratory signs of hospitalised severe COVID-19 patients.

## Patients and methods

### Study design

This study was conducted as a single-blind, two-arm parallel, randomised controlled trial from April 20, 2020 to June 20, 2020. We enrolled 68 subjects from the Imam Khomeini Hospital, Tehran University of Medical Sciences (Tehran, Iran) and Khorshid Hospital, Isfahan University of Medical Sciences (Isfahan, Iran). The study protocol was written and mediated in accordance with the CONSORT (Consolidated Standards of Reporting Trials) statement [14] and the study was registered at the Iranian Registry of Clinical Trials on April 15, 2020 with identifier IRCT20200404046947N1. Since there are no published clinical trials on the effect of methylprednisolone in patients with COVID-19, the minimum sample size was estimated as 60 plus 10% potential missing data based on the effect size of methylprednisolone on pulmonary and lung function diseases [15–17].

This trial was performed based on the Declaration of Helsinki guidelines and was approved by the Ethics Committee of Tehran University of Medical Sciences (approval IR.TUMS.VCR.REC.1399.054).

### Patients

The diagnosis of COVID-19 in subjects was performed based on the following criteria: 1) identification of SARS-CoV-2 via reverse transcription-PCR in nasopharyngeal swab or sputum samples and 2) abnormal computed tomography (CT) scan findings (bilateral, subpleural, peripheral ground-glass opacities) with blood arterial oxygen saturation measured by pulse oximetry (*S*_pO_2__) <90% at rest. The early pulmonary phase was defined as the start of pulmonary involvement, including hypoxia (*S*_pO_2__ <93%), tachypnoea (respiratory rate >18 breaths·min^−1^) and little dyspnoea, and based on CT scan findings. All patients signed informed consent before being enrolled in the study.

#### Inclusion criteria

**FIGURE 1:**
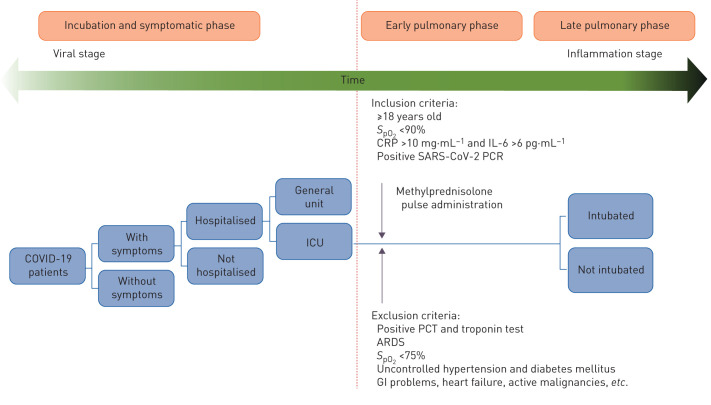
Appropriate time for methylprednisolone administration and inclusion/exclusion criteria of the patients. Patients in the intervention group received methylprednisolone pulse (intravenous injection, 250 mg·day^−1^ for 3 days) at the early pulmonary phase of the disease before connection to the ventilator and intubation. ICU: intensive care unit; *S*_pO_2__: arterial oxygen saturation measured by pulse oximetry; CRP: C-reactive protein; IL: interleukin; SARS-CoV-2: severe acute respiratory syndrome coronavirus 2; PCT: pro-calcitonin; ARDS: acute respiratory distress syndrome; GI: gastrointestinal.

Patients were included in the study if they met the following criteria: 1) aged ≥18 years, 2) confirmed COVID-19 with *S*_pO_2__ <90%, elevated C-reactive protein (CRP) (>10 mg·L^−1^) and interleukin (IL)-6 (>6 pg·mL^−1^) at the early pulmonary phase of disease before connecting to the ventilator and intubation, and 3) agreed to give informed consent (figure 1).

#### Exclusion criteria

Individuals were excluded from the study if they met the following criteria: 1) intolerant or allergic to any therapeutic agents used in this research, 2) pregnant or lactating females, and 3) *S*_pO_2__ <75%, positive pro-calcitonin (PCT) and troponin test, ARDS, uncontrolled hypertension, uncontrolled diabetes mellitus, gastrointestinal problems or gastrointestinal bleeding history, heart failure, active malignancies or received any immunosuppressor agents.

### Randomisation and masking

Once eligibility had been confirmed (24–48 h after hospitalisation), the patients were randomly allocated to control (n=34) and intervention (n=34) groups in a 1:1 ratio by the block randomisation method. Patients were allocated to either receive methylprednisolone pulse (intravenous injection, 250 mg·day^−1^ for 3 days) or not receive methylprednisolone/other glucocorticoids. All patients received standard care (hydroxychloroquine sulfate, lopinavir and naproxen) for COVID-19 according to the protocol for diagnosis and treatment of COVID-19 in Iran. Patients were blinded to the treatment group. Physicians and clinician teams knew about the medicine and intervention groups. Due to the emergency nature of this trial, placebos of methylprednisolone were not prepared.

### Procedures and outcomes

Clinical and demographic characteristics of the study participants were obtained before enrolment in the study. All patients were followed-up from day 0 to day 3, improvement, hospital discharge or death and 1 week after hospital discharge, which was scheduled at three or four consecutive visit points. Clinical signs of the patients including heart rate, body temperature, blood pressure, *S*_pO_2__, dyspnoea, cough, gastrointestinal involvement symptoms, myalgia, chest pain and Borg score were assessed before and after treatment (by 3 days of treatment and discharge time). The need for oxygen therapy (nasal cannula, mask oxygen, reservoir mask, noninvasive ventilation (NIV) and invasive ventilation) was recorded before and after treatment (by 3 days of treatment and discharge time). CT scan findings were also assessed before and 1 week after treatment only if patients agreed to give informed consent. Other clinical outcomes, including mortality rate, duration of hospitalisation in improved patients and the time from the initiation of treatment to death, were assessed in each group. Laboratory test results, including complete blood count, CRP, erythrocyte sedimentation rate, venous blood gas (VBG) analysis (including pH, bicarbonate (HCO_3_) and partial pressure of carbon dioxide (*P*CO_2_)), IL-6, ferritin, troponin, D-dimer, lactate dehydrogenase (LDH) and creatine phosphokinase (CPK), were recorded before and after treatment (by 3 days of treatment and discharge time). Clinical signs of the improved patients, including cough, gastrointestinal symptoms, myalgia, chest pain and Borg score, were assessed 1 week after discharge time.

All data were considered during the study and follow-up time, and recorded on case report forms and an Excel (Microsoft, Redmond, VA, USA) database. The primary end-points were the time of clinical improvement and time of discharge from the hospital or death, whichever came first. Hospital discharge was determined according to the patient's clinical and laboratory findings. Improvement was defined as Borg score >3, improved dyspnoea, no fever for 72 h, *S*_pO_2__ >93%, tolerated oral regime, normal urinary output and reduced CRP level without any treatment side-effects.

### Adverse events

All undesirable effects (adverse events) experienced by patients during the study, whether or not related to methylprednisolone treatment, were defined and recorded.

### Statistical analysis

Data are presented as means with standard deviations for continuous variables, unless otherwise stated. Categorical variables are presented as numbers and percentages. The Kolmogorov–Smirnov normality test was performed on all data. Repeated measures ANOVA was used for comparison of the trends over time between both groups for each studied variable. Moreover, the t-test (parametric) or Mann–Whitney test (nonparametric) was used to test for statistical differences (two-tailed) between two independent groups. The paired t-test (parametric) or Wilcoxon signed-rank test (nonparametric) was used to test for statistical differences between two time-points in each of the intervention groups. Two-sided Chi-squared/Fisher's exact tests were used to assess the associations between intervention groups and the categorical variables. Kaplan–Meier survival curve analysis and the log-rank test were used to analyse time to death between both intervention groups. After analysing the baseline data, using the intention-to-treat (ITT) test, multiple imputations were conducted by an expectation maximisation algorithm in order to make an unbiased comparison between intervention groups in handling missing data. The false discovery rate was corrected using the Benjamini–Hochberg correction method for multiple comparisons. All statistical analysis was performed using Stata version 11.2 (StataCorp, College Station, TX, USA). Statistical significance was considered at p<005.

## Results

### Patients

**FIGURE 2:**
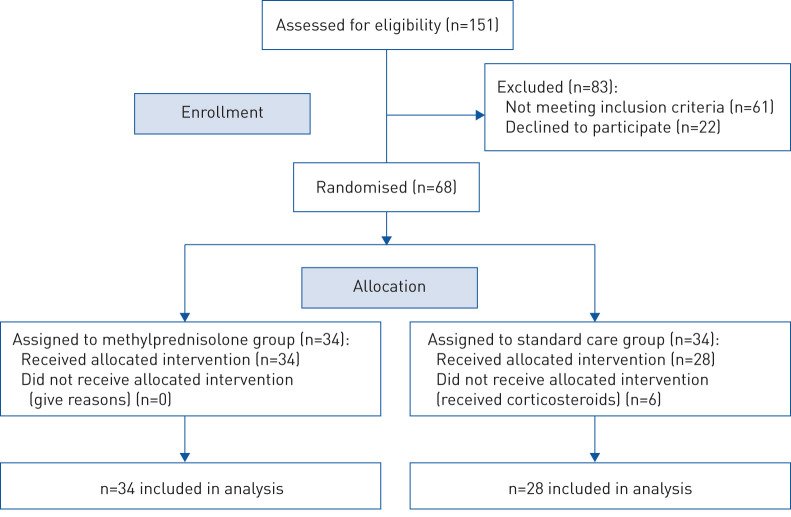
Randomisation, enrolment and treatment assignment.

Of the 68 patients who underwent randomisation, 34 patients were assigned to receive standard care and methylprednisolone, and 34 patients were assigned to standard care alone. In the standard care group, six patients received corticosteroids by the attending physician before the treatment and were excluded from the overall analysis. Based on the per-protocol analysis, the results for the outcomes did not significantly differ from the results of the ITT analysis. Randomisation, enrolment and treatment assignment are described in figure 2. The age of patients was 58.5±16.6 years (23 (37.1%) females and 39 (62.9%) males). 22 patients (35.5%) had a respiratory rate >24 breaths·min^−1^ and 13 patients (21.0%) had a heart rate >100 beats·min^−1^. Coexisting conditions and demographic and clinical characteristics in each group are shown in table 1. Respiratory rate and heart rate levels were significantly higher in the methylprednisolone group. Except for diabetic comorbidity, which was significantly higher in the standard care group, there were no major between-group differences in demographic and clinical characteristics at enrolment. The median±range interval between disease symptom onset and hospitalisation was 6.8±3.0 days. *S*_pO_2__ and Borg score of patients were 82.7±5.3% and 7.4±2.1, respectively, at baseline. The majority of patients had 30–50% (24 (38.7%)) or 50–70% (19 (30.6%)) pulmonary involvement and all patients were receiving oxygen support. Table 2 shows status and pulmonary involvement level at baseline for the patients in each group. Except for the difference in the pulmonary involvement zone, there were no between-group differences in status and pulmonary involvement at enrolment.

**TABLE 1:** Baseline demographic and clinical characteristics of patients at baseline

	**Total**	**Methylprednisolone**	**Standard care**	**p-value**
**Subjects**	62	34	28	
**Age years**	58.5±16.6	55.8±16.4	61.7±16.6	0.193
**Sex**				0.205
Male	39 (62.9)	24 (70.6)	15 (53.6)	
Female	23 (37.1)	10 (29.4)	13 (46.4)	
**Coexisting conditions**				
Diabetes	22 (35.5)	8 (23.5)	14 (50.0)	0.040*
Hypothyroidism	4 (6.5)	4 (11.8)	0	0.118
Cancer	3 (4. 8)	1 (2.9)	2 (7.1)	0.590
Respiratory disorder	6 (9.7)	3 (8.8)	3 (10.7)	0.838
Renal disorder	7 (11.3)	3 (8.8)	4 (14.3)	0.532
Cardiovascular disorder	11 (17.7)	6 (17.6)	5 (17.9)	0.966
Hypertension	20 (32.3)	10 (29.4)	10 (35.7)	0.666
Autoimmune and neurodegenerative diseases	5 (8.1)	2 (5.9)	3 (10.7)	0.654
**Body temperature C**	37.3±0.8	37.4±0.9	37.2±0.8	0.356
**Respiratory rate breaths·min^−1^**	22.6±4.6	23.7±4.6	21.4±4.5	0.048*
**Respiratory rate >24 breaths·min^−1^**	22 (35.5)	16 (47.1)	6 (21.4)	0.028*
**Heart rate beats·min^−1^**	89.7±14.8	93.9±13.2	84.6±15.3	0.010*
**Heart rate >100 beats·min^−1^**	13 (21.0)	10 (29.4)	3 (10.7)	0.062
**Systolic blood pressure mmHg**	122.6±14.9	121.4±15.3	124.0±14.7	0.563
**Systolic blood pressure <100 mmHg**	0	0	0	NA
**Diastolic blood pressure mmHg**	75.6±8.6	76.1±9.4	75.3±7.7	0.643
**Fever**	32 (51.6)	17 (50.0)	15 (53.6)	0.891
**Dyspnoea**	39 (62.9)	23 (67.6)	16 (57.1)	0.310
**GI symptoms**	29 (46.8)	16 (47.1)	13 (46.4)	0.961
**Myalgia**	32 (51.6)	17 (50.0)	15 (53.6)	0.779
**Headache**	8 (12.9)	3 (8.8)	5 (17.9)	0.453
**Cough**	38 (61.3)	23 (67.6)	15 (53.6)	0.498
**Weakness**	17 (27.4)	9 (26.5)	8 (28.6)	0.921
**WBC count ×10^3^ mg·L^−1^**	7.6±3.9	7.7±3.3	7.4±4.6	0.866
4–10	47 (75.8)	25 (73.5)	22 (78.6)	0.811
<4	7 (11.3)	4 (11.8)	3 (10.7)	0.893
>10	7 (11.3)	5 (14.7)	2 (7.1)	0.697
**Lymphocyte count mg·L^−1^**	1169.0±597.1	1167.7±580.7	1170.7±632.4	0.866
800–5000	42 (67.7)	25 (73.5)	17 (60.7)	0.643
<800	16 (25.8)	9 (26.5)	7 (25.0)	0.811
>5000	0	0	0	NA
**Platelet count ×10^3^ mg·L^−1^**	219.9±106.8	203.8±69.7	241.0±140.4	0.158
<150	10 (16.1)	6 (17.6)	4 (14.3)	0.426
150–450	49 (79.0)	28 (82.4)	21 (75.0)	0.513
>450	1 (1.6)	0	1 (3.8)	NA
**Haemoglobin g·dL^−1^**	13.7±4.2	13.0±1.7	14.7±6.0	0.102
**VBG analysis**				
pH	7.40±0.04	7.42±0.05	7.39±0.03	0.046*
HCO_3_ mEq·L^−1^	24.4±5.6	25.5±4.7	22.9±6.4	0.096
*P*_CO_2__ mmHg	37.9±10.1	39.7±11.1	35.4±8.1	0.156
**CRP mg·L^−1^**	96.1±75.2	99.1±80.0	92.6±70.3	0.707
**ESR mm·h^−1^**	61.6±29.8	64.2±27.1	57.4±32.8	0.382
**IL-6 pg·mL^−1^**	77.4±76.1	76.3±85.0	79.3±60.9	0.894
**D-dimer ng·mL^−1^**	2053.3±2363.7	2573.5±2667.7	1391.3±1750.9	0.057
**Ferritin ng·mL^−1^**	750.9±496.6	807.9±521.0	676.5±463.5	0.345
**LDH U·L^−1^**	658.5±235.6	672.1±285.5	644.9±179.3	0.728
**CPK U·L^−1^**	176.3±168.9	158.9±151.7	213.7±209.0	0.491
**Troponin negative**	63 (100)	34 (100)	28 (100)	NA
**SARS-CoV-2 PCR positive**	63 (100)	34 (100)	28 (100)	NA
**PCT negative**	63 (100)	34 (100)	28 (100)	NA

Data are presented as n, mean±sd or n (%), unless otherwise stated. GI: gastrointestinal; WBC: white blood cell; VBG: venous blood gas; *P*_CO_2__: partial pressure of carbon dioxide; CRP: C-reactive protein; ESR: erythrocyte sedimentation rate; LDH: lactate dehydrogenase; CPK: creatine phosphokinase; PCT: pro-calcitonin; NA: not available. *: p<0.05.

**TABLE 2:** Status and pulmonary involvement level of patients at baseline

	**Total**	**Methylprednisolone**	**Standard care**	**p-value**
**Subjects**	62	34	28	
**Time from illness onset to hospitalisation** **days^#^**	6.8±3.0	6.7±2.9	6.9±3.1	0.814
**Borg score**	7.4±2.1	7.7±1.7	7.1±2.6	0.182
***S*_pO_2__ %**	82.7±5.3	82.0±5.8	83.6±4.7	0.267
**Need for oxygen therapy**	62 (100)	34 (100)	28 (100)	0.460
**Type of oxygen therapy**				
Nasal cannula	13 (21.0)	4 (11.8)	9 (32.1)	0.194
Simple mask	7 (11.3)	5 (14.7)	2 (7.1)	
Reservoir mask	18 (29.0)	12 (35.3)	6 (21.4)	
NIV	23 (37.1)	13 (38.2)	10 (35.7)	
**Ground-glass opacity**	53 (85.5)	30 (88.2)	23 (82.1)	0.334
Unilateral	0	0	0	NA
Bilateral	53 (100)	30 (100)	23 (100)	NA
**Consolidation positive**	43 (69.3)	23 (67.6)	20 (71.4)	0.911
Unilateral	6 (14.0)	3 (13.0)	3 (15.0)	0.977
Bilateral	37 (86.0)	20 (87.0)	17 (85.0)	
**Pulmonary involvement**				
A (<10%)	0	0	0	0.050
B (10–30%)	7 (11.3)	1 (2.9)	6 (21.4)	
C (30–50%)	24 (38.7)	13 (38.2)	11 (39.3)	
D (50–70%)	19 (30.6)	11 (32.4)	8 (28.6)	
E (>70%)	12 (19.4)	9 (26.5)	3 (10.7)	
**Pulmonary involvement zone**				
All	28 (44.4)	20 (58.8)	8 (28.6)	0.031*
Upper	5 (8.1)	3 (8.8)	2 (7.1)	0.728
Lower	23 (37.1)	10 (29.4)	13 (46.4)	0.104
Middle	18 (29.0)	11 (32.4)	7 (25.0)	0.649

Data are presented as n, mean±sd or n (%), unless otherwise stated. *S*_pO_2__: arterial oxygen saturation measured by pulse oximetry; NIV: noninvasive ventilation; NA: not available. ^#^: median±range. *: p<0.05.

### Primary outcomes

Patients in the methylprednisolone group had a significantly reduced median±range time to event (discharge or death) compared with patients in the standard care group (11.62±4.81 *versus* 17.61±9.84 days; p=0.006). In addition, median±range time to improvement was significantly lower in the methylprednisolone group (11.84±4.88 *versus* 16.44±6.93 days; p=0.011) compared with the standard care group and methylprednisolone treatment was related to the shorter time to event in patients (table 3). The percentage of improved patients was higher in the methylprednisolone group than in the standard care group (32 (94.1%) *versus* 16 (57.1%)) and the mortality rate was significantly lower in the methylprednisolone group (2 (5.9%) *versus* 12 (42.9%); p<0.001).

**TABLE 3:** Primary outcomes in the methylprednisolone and standard care groups

	**Methylprednisolone**	**Standard care**	**p-value**
**Subjects**	34	28	
**Time to event (discharge or death)** **days^#^**	11.62±4.81	17.61±9.84	0.006*
**Time to improvement days^#^**	11.84±4.88	16.44±6.93	0.011*
**Outcome**			<0.001*
Recovery	32 (94.1)	16 (57.1)	
Death	2 (5.9)	12 (42.9)	

Data are presented as n or n (%), unless otherwise stated. ^#^: median±range. *: p<0.05.

**FIGURE 3:**
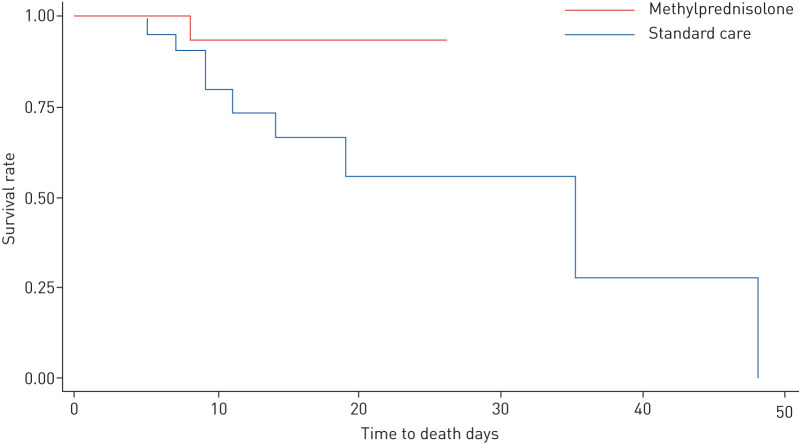
Kaplan–Meier estimator of survival rate between the methylprednisolone and standard care groups. Log-rank test: p<0.001; hazard ratio 0.293 (95% CI 0.154–0.556).

Using the Kaplan–Meier estimator of time to death, we demonstrated that patients in the methylprednisolone igroup had a significantly increased survival time compared with patients in the standard care group (log-rank test: p<0.001; hazard ratio 0.293, 95% CI 0.154–0.556) (figure 3).

Incidence of death was significantly lower in patients receiving NIV, reservoir mask and nasal cannula support in the methylprednisolone group (7.7%, 8.3% and 0%, respectively) compared with the standard care group (60%, 57.1% and 22%, respectively) (supplementary figure S1). CT scan findings from all of the deceased patients in the methylprednisolone group (n=2) and 75% of the patients in the standard care group (n=9) showed bilateral ground-glass opacities at enrolment.

### Secondary outcomes

*S*_pO_2__ and Borg score were significantly improved after 3 days of treatment and at discharge time in the methylprednisolone group, whereas *S*_pO_2__ was significantly decreased in the standard care group after 3 days of treatment and the increase of *S*_pO_2__ at discharge time was not significant in this group. The Borg score of patients did not change after 3 days of treatment in the standard care group and a significant decrease was only observed at discharge time in this group (table 4).

**TABLE 4:** Clinical characteristics of patients before and after treatment (analysed by repeated measures ANOVA)

	**Before treatment**		**After treatment (day 3)**				**After treatment (discharge or death)**			
	**Methylprednisolone**	**Standard care**	**Methylprednisolone**	**p-value**	**Standard care**	**p-value**	**Methylprednisolone**	**p-value**	**Standard care**	**p-value**
**Borg score**	7.7±1.7	7.1±2.6	4.8±2.2	<0.001*	6.2±3.4	0.154	1.7±0.8	<0.001*	1.8±1.8	0.001*
***S*_pO_2__ %**	82.1±5.7	83.6±4.7	88.1±6.1	<0.001*	80.5±8.3	0.03*	92.7±2.6	<0.001*	89.0±5.0	0.08
**Systolic blood pressure mmHg**	121.4±15.2	124.0±14.6	127.6±15.3	0.049*	122.8±20.0	0.544	126±14.0	0.009*	124.5±35.3	0.75
**Diastolic blood pressure mmHg**	76.1±9.4	75.3±7.7	78.0±8.8	0.28	74.0±14.2	0.76	74.7±9.0	0.54	74.0±8.2	0.33
**Heart rate beats·min^−1^**	93.9±13.2	84.6±15.3	82.0±11.9	<0.001*	84.7±13.8	0.78	81.2±7.9	0.003*	85.9±17.7	0.41
**Respiratory rate breaths·min^−1^**	23.7±4.6	21.4±4.5	20.8±3.1	0.001*	23.1±5.1	0.047*	18.6±2.4	<0.001*	21.0±3.8	0.89
**Body temperature °C**	37.3±0.9	37.2±0.8	36.7±0.4	0.001*	36.8±0.6	0.09	36.7±0.4	0.008*	36.8±0.3	0.29
**GI symptoms**	16 (47.1)	13 (46.4)	2 (5.9)	<0.001*	5 (17.9)	0.18	1 (3.1)	<0.001*	1 (6.3)	0.014*
**Myalgia**	17 (50.0)	15 (53.6)	4 (11.8)	0.001*	5 (17.9)	0.025*	0	<0.001*	1 (6.3)	0.025*
**Chest pain**	7 (20.6)	5 (17.9)	2 (5.9)	0.025*	2 (7.1)	0.16	1 (3.1)	0.014*	1 (6.3)	0.32
**Cough**	23 (67.6)	16 (57.1)	12 (35.3)	0.003*	12 (42.9)	0.16	6 (18.8)	<0.001*	6 (37.5)	0.32
**Need for oxygen therapy**	34 (100)	28 (100)	28 (82.4)	0.025*	26 (92.8)	0.56	6 (18.8)	<0.001*	6 (37.5)	0.003*

Data are presented as mean±sd or n (%), unless otherwise stated. *S*_pO_2__: arterial oxygen saturation measured by pulse oximetry; GI: gastrointestinal; NA: not available. *: p<0.05.

Heart rate and temperature were significantly decreased after 3 days of treatment and at discharge time only in the methylprednisolone group. Respiratory rate was also significantly reduced in the methylprednisolone group after treatment, whereas it was significantly increased in the standard care group after 3 days of treatment. Clinical characteristics of patients, including gastrointestinal symptoms, myalgia, chest pain and cough, were significantly improved in the methylprednisolone group after 3 days of treatment and at discharge time; however, chest pain and cough did not change significantly in the standard care group after treatment. Clinical characteristics of patients before and after treatment are shown in table 4.

Six out of 34 patients, by 3 days of treatment, and 26 out of 32 patients, at discharge time, did not need oxygen support in the methylprednisolone group. In the standard care group, two out of 28 patients, by 3 days of treatment, and 10 out of 16 patients, at discharge time, did not need oxygen support (table 4). 19 out of 34 patients (55.8%) showed improvement and three out of 34 patients (8.8%) showed worsening in oxygen support status by 3 days of treatment in the methylprednisolone group, whereas six out of 28 patients (21.4%) showed improvement and 14 out of 28 patients (50%) showed worsening in the standard care group (supplementary figure S1). Patient status regarding oxygen support before and after treatment and the main outcome of each group are shown in supplementary figure S1.

To assess the percent of pulmonary involvement of patients in the methylprednisolone group, CT was performed at discharge time on 11 out of 31 discharged patients who agreed to give informed consent. The results showed that after treatment pulmonary involvement was improved 20–30% in eight out of the 11 patients and 50–60% in three out of the 11 patients (supplementary figure S2). CT scan findings and improvement in pulmonary involvement after treatment in a patient in the methylprednisolone group are shown in supplementary figure S3.

### Laboratory findings

White blood cell count was significantly increased after 3 days of treatment and at discharge time in the methylprednisolone group, whereas it was not changed in the standard care group by 3 days of treatment and was only significantly increased at discharge time. Haemoglobin level and lymphocyte count were significantly decreased in the methylprednisolone group after 3 days of treatment and were restored at discharge time. We did not find a significant change in haemoglobin level and lymphocyte count in the standard care group before and after treatment. Although platelet count remained unchanged during treatment in the standard care group, it was significantly increased in the methylprednisolone group after treatment. VBG pH, HCO_3_ and *P*CO_2_ levels remained unchanged until discharge time in the methylprednisolone group, whereas VBG HCO_3_ and *P*CO_2_ levels were increased in the standard care group after treatment. Decreases in CRP and IL-6 levels were only shown in the methylprednisolone group after treatment. D-dimer, ferritin, LDH and CPK levels did not show any significant changes before and after treatment in either group of patients (table 5).

**TABLE 5:** Laboratory findings of patients before and after treatment (analysed by repeated measures ANOVA)

	**Before treatment**		**After treatment (day 3)**				**After treatment (discharge or death)**			
	**Methylprednisolone**	**Standard care**	**Methylprednisolone**	**p-value**	**Standard care**	**p-value**	**Methylprednisolone**	**p-value**	**Standard care**	**p-value**
**WBC count ×10^3^ mg·L^−1^**	7.7±3.3	7.4±4.6	9.5±3.8	0.008*	8.1±4.6	0.36	10.5±4.9	0.002*	9.6±5.6	0.03*
**Lymphocyte count mg·L^−1^**	1167±580	1171±632	804±345	<0.001*	1212±689	0.95	1085±667	0.77	1315±813	0.72
**Haemoglobin g·dL^−1^**	13.0±1.7	14.7±6.0	12.1±2.1	0.007*	15.0±7.8	0.79	12.6±1.7	0.59	10.7±1.8	0.13
**Platelet count ×10^3^ mg·L^−1^**	204±70	241±140	271±93	<0.001*	243±82	0.87	252±90	0.02*	193±127	0.88
**VBG analysis**										
pH	7.42±0.05	7.39±0.03	7.42±0.04	0.40	7.40±0.10	0.88	7.40±0.08	0.35	7.40±0.08	0.06
HCO_3_ mEq·L^−1^	25.5±4.7	22.9±6.4	26.4±4.6	0.73	27.4±1.6	0.011*	27.4±8.9	0.48	31.3±4.4	0.001*
*P*_CO_2__ mmHg	39.7±11.1	35.4±8.1	40.9±10.7	0.85	50.8±16.2	0.017*	45.4±16.4	0.39	NA	NA
**CRP mg·L^−1^**	99.1±80.0	92.6±70.3	40.8±30.6	<0.001*	91.9±68.0	0.66	30.6±23.1	0.001*	77±83.0	0.20
**ESR mm·h^−1^**	64.2±27.1	57.4±32.8	50.9±35.3	0.11	57.0±33.6	0.86	55.7±22.6	0.09	80.0±25.7	0.77
**IL-6 pg·mL^−1^**	76.3±85.0	79.3±60.9	15.9±22.9	<0.001*	32.5±6.5	0.160	3.9±9.1	0.001*	NA	NA
**D-dimer ng·mL^−1^**	2573±2668	1391±1751	2155±1441	0.41	2131±2252	0.25	1762±830	0.16	NA	NA
**Ferritin ng·mL^−1^**	808±521	677±463	766±400	0.17	777±467	0.62	275±303	0.18	476±219	0.28
**LDH U·L^−1^**	672±286	645±179	897±558	0.74	706±453	0.91	633±153	0.49	NA	NA
**CPK U·L^−1^**	159±152	214±209	249±154	0.78	NA	NA	85±203	0.81	NA	NA

Data are presented as mean±sd, unless otherwise stated. WBC: white blood cell; VBG: venous blood gas; *P*_CO_2__: partial pressure of carbon dioxide; CRP: C-reactive protein; ESR: erythrocyte sedimentation rate; IL: interleukin; LDH: lactate dehydrogenase; CPK: creatine phosphokinase; NA: not available. *: p<0.05.

### Safety and follow-up

Two patients (5.8%) in the methylprednisolone group and two patients (7.1%) in the standard care group showed severe adverse events between initiation of treatment and the end of the study. There was one infection and one oedema adverse event in the methylprednisolone group, and two shock adverse events in the standard care group (supplementary table S1). All events and deaths during the study were judged by the site investigators to be unrelated to the intervention. In addition, no psychiatric or delirium events were detected in patients. Following the use of high-dose corticosteroids, most of the patients required insulin due to their known or hidden diabetes, and the insulin requirement was increased in the methylprednisolone group, especially in diabetic and overweight patients. However, the insulin requirement level was controlled by physicians and returned to normal at discharge time and there were no adverse events according to uncontrolled diabetes in patients. Borg score and clinical characteristics of recovered patients (n=48) were assessed 1 week after discharge time. Borg score was significantly diminished 1 week after discharge time in both groups. None of the patients in the methylprednisolone group had gastrointestinal symptoms, myalgia or chest pain after discharge. Two patients in the standard care group still had gastrointestinal symptoms and myalgia after discharge. Six out of 32 patients (18.8%) in the methylprednisolone and three out of 16 patients (18.8%) in the standard care group still had cough 1 week after discharge (supplementary table S2).

## Discussion

The current study is the first randomised controlled trial to evaluate changes in clinical symptoms and laboratory signs of COVID-19 patients by methylprednisolone therapy. The study found that methylprednisolone pulse administration at the beginning of the early pulmonary phase of illness remarkably decreased the mortality rate and improved pulmonary involvement, *S*_pO_2__ and inflammatory markers in COVID-19 patients. Given the increased incidence and mortality of COVID-19 across the world, the helpful and effective treatment for patients in the early pulmonary phase is still of paramount importance. There have been some reports surrounding beneficial [11] or harmful evidence [18, 19] of corticosteroid therapy during previous SARS and MERS outbreaks, but the reports are not conclusive [20]. However, the clinical evidence for the efficacy of receiving corticosteroids in COVID-19 patients and the time for administration are undetermined.

The current study enrolled a severely ill population of COVID-19 patients in the early pulmonary phase (not intubated). The mortality rate was observed to be significantly lower among patients treated with methylprednisolone than patients treated with standard care. 94.1% of patients in the methylprednisolone group had recovered by a median duration of 11.8 days. However, only 57.1% of patients in the standard care group had recovered by a median duration of 16.4 days. Methylprednisolone treatment was related to the shorter time to event in patients and survival analysis showed that the patients in the methylprednisolone group had a significantly decreased death hazard rate compared with the patients in the standard care group.

The effect of dexamethasone on the clinical symptoms of hospitalised COVID-19 patients was studied in the clinical trial by the RECOVERY Collaborative Group [21]. A total of 2104 patients received dexamethasone and 4321 received standard care. Their results showed that the incidence of death was significantly lower in patients receiving oxygen support and invasive mechanical ventilation. In our study, all patients received oxygen support and none of them received mechanical ventilation; however, in line with the RECOVERY trial, the incidence of death was significantly lower in patients receiving NIV and reservoir mask support in the methylprednisolone group (7.7% and 8.3%, respectively) compared with the standard care group (60.0% and 57.1%, respectively). In addition, some observational studies report recent clinical findings on the administration of corticosteroids in the treatment of COVID-19 [22]. Some studies did not find significant benefits of corticosteroid admission and reported that pulmonary involvements caused by SARS-CoV-2 were not inhibited by corticosteroid treatment [23–25]. However, it was also reported that the administration of corticosteroid for patients with ARDS resulted in a reduced risk of death [26]. The observed differences could be due to the differences in the amount and duration of treatment, small sample size, age of patients, and severity of the disease. The clinical and laboratory characteristics and pulmonary involvements of patients were not fully determined and reported in those observational studies. It seems that the administration time and pulmonary phase of patients are key factors in corticosteroid treatment efficacy.

In our study, patients in the methylprednisolone group had a faster improvement in *S*_pO_2__, Borg score and dyspnoea. Improvement and worsening in oxygen support status were observed in 55.8% and 8.8%, respectively, of patients in the methylprednisolone group by day 3 of treatment, whereas in the standard care group only 21.4% of patients showed improvement in oxygen support and 50% showed worsening. Our results show that patients in the methylprednisolone group were less likely to receive invasive ventilation. Only 8.8% of patients in the methylprednisolone group received invasive ventilation, whereas in the standard care group, 32.1% of patients received mechanical ventilation after treatment. In line with our results, in a cohort study by Wang *et al.* [27], it was demonstrated that patients with methylprednisolone treatment had a faster improvement of *S*_pO_2__, decrease in CRP and IL-6 levels, and were less likely to receive invasive ventilation. However, Wang *et al.* [27] did not observe significant differences in mortality rate between groups.

Our results also showed that corticosteroid therapy can improve ventilation in patients. VBG analysis showed an increase in HCO_3_ and *P*CO_2_ levels in the standard care group, which can indicate respiratory acidosis and decreased ventilation in patients [28], whereas VBG markers did not change significantly in the methylprednisolone group.

The clinical characteristics of patients, including heart rate, respiratory rate and temperature, were also significantly improved in the methylprednisolone group, whereas they did not change or worsen in the standard care group during treatment. Gastrointestinal symptoms and myalgia were improved in patients from both groups, whereas chest pain and cough were only significantly improved in the methylprednisolone group. Intravenous methylprednisolone administration increased blood pressure in patients, which is due to the hypertensive side-effects of glucocorticoids [29].

It has been demonstrated that elevated serum levels of IL-6 and CRP as inflammatory markers are associated with the severity of COVID-19 and can be used as a predictive factor for disease risk [30]. Patients included in this trial had increased CRP and IL-6 serum levels at enrolment. A significant decrease in the serum levels of these inflammatory markers was shown only in the methylprednisolone group after treatment.

Previous studies reported that corticosteroid administration can increase the risk of post-treatment infection in viral disease; however, in our study the incidence of nosocomial infections was very low in both the methylprednisolone and standard care groups. Improved patients were followed-up for 7 days after treatment and clinical symptoms remained unchanged. We will continue to follow-up the patients, and CT scans, spirometry and pulse oximetry will be performed for 6 weeks after improvement to evaluate their long-term prognosis.

### Conclusions

In this study, we assessed the intravenous methylprednisolone effect on the treatment of patients with severe COVID-19. Clinical data showed that methylprednisolone administration at the beginning of the early pulmonary phase of illness remarkably improved pulmonary involvement, oxygen saturation, dyspnoea, heart rate, respiratory rate, temperature and inflammatory markers (*e.g.* CRP and IL-6 serum levels) in patients, suggesting that methylprednisolone could be an efficient therapeutic agent for hospitalised severe COVID-19 patients at the pulmonary phase. Unfortunately, we could not collect viral load data to assess the effects of methylprednisolone on viral load changes between baseline and discharge time. There are several other limitations in this study, including the possible existence of bias, single-blind design of the study, lack of follow-up to identify late adverse events (*e.g.* hip osteonecrosis or tuberculosis re-activation) and limited sample size. Further studies need to be undertaken.

## Supplementary material

10.1183/13993003.02808-2020.Supp1 **Please note:** supplementary material is not edited by the Editorial Office, and is uploaded as it has been supplied by the author.
Supplementary figure S1 ERJ-02808-2020.Figure_S1
Supplementary figure S2 ERJ-02808-2020.Figure_S2
Supplementary figure S3 ERJ-02808-2020.Figure_S3
Supplementary table S1 ERJ-02808-2020.Table_S1
Supplementary table S2 ERJ-02808-2020.Table_S2

## Shareable PDF

10.1183/13993003.02808-2020.Shareable1 This one-page PDF can be shared freely online.
Shareable PDF ERJ-02808-2020.Shareable
