# Supplementary material for: Intravenous methylprednisolone pulse as a treatment for hospitalised severe COVID-19 patients: results from a randomised controlled clinical trial
Source: Eur Respir J. 2020 Dec 24;56(6):2002808. doi: 10.1183/13993003.02808-2020 (PMC7758541; doi:10.1183/13993003.02808-2020)
Supplement: Supplementary file 1 [file ERJ-02808-2020.Figure_S1.pdf]

		Number of patients in oxygen support group at baseline (%)									
		Methylprednisolone group					Standard care group				
		NIV (13)	Reserve Mask (12)	Simple Mask (5)	Nasal Cannula (4)	Negative (0)	NIV (10)	Reserve Mask (7)	Simple Mask (2)	Nasal Cannula (9)	Negative (0)
Number of patients in oxygen support group after 3 days of treatment (%)	Invasive Ventilation	2 (23.1%)	0	0	0	0	5 (50.0%)	3 (42.8%)	0	1 (11.1%)	0
	NIV	3 (23.1%)	0	0	0	0	3 (30.0%)	1 (14.3%)	1 (50.0%)	1 (11.1%)	0
	Reserve Mask	5 (38.5%)	5 (41.7%)	0	0	0	1 (10.0%)	0	0	2 (22.2%)	0
	Simple Mask	1 (7.7%)	1 (8.3%)	1 20.0%)	0	0	0	0	1 (50.0%)	0	0
	Nasal Cannula	2 (15.4%)	3 (25.0%)	2 (40.0%)	2 (50.0%)	0	1 (10.0%)	3 (42.8%)	0	4 (44.4%)	0
	negative	0	1 (8.3%)	2 (40.0%)	2 (50.0%)	0	0	0	0	1 (11.1%)	0
Outcome	Discharged	12 (84.6%)	11 (91.7%)	5 (100%)	4 (100%)	0	4 (40.0%)	3 (42.8%)	2 (100%)	7 (77.7%)	0
	Death	1 (7.7%)	1 (8.3%)	0	0	0	6 (60.0%)	4 (57.1%)	0	2 (22.2%)	0

Supplementary Figure 1. The patient's status regarding their oxygen supports before and after treatment and the main outcome of each group. For each oxygen-support category (invasive ventilation, noninvasive ventilation (NIV), reserve mask, simple mask, and nasal cannula), percentages were calculated with the number of patients at baseline and after 3 days of treatment in both groups. Improvement (green cells), no change (blue), and worsening (orange) in oxygen-support status are shown.
