# Supplementary material for: Intravenous methylprednisolone pulse as a treatment for hospitalised severe COVID-19 patients: results from a randomised controlled clinical trial
Source: Eur Respir J. 2020 Dec 24;56(6):2002808. doi: 10.1183/13993003.02808-2020 (PMC7758541; doi:10.1183/13993003.02808-2020)
Supplement: Supplementary file 2 [file ERJ-02808-2020.Figure_S2.pdf]

		Number of patients in pulmonary involvements group before treatment				
		Methylprednisolone group (11)				
		E (>70%)	D (50-70%)	C (30-50%)	B (10-30%)	A (<10%)
Number of patients in pulmonary involvements group at discharge time	E (>70%)	0	0	0	0	0
	D (50-70%)	0	0	0	0	0
	C (30-50%)	3	3	0	0	0
	B (10-30%)	0	0	4	0	0
	A (<10%)	0	0	0	1	0

Supplementary Figure 2. Number of patients in different pulmonary involvement groups before and after treatment in the methylprednisolone group. For each pulmonary involvement category (A-E), the number of patients at baseline and at discharge time in the methylprednisolone group has been shown. Improvement (green cells), no change (blue), and worsening (orange) in pulmonary involvements status are shown.
