# Supplementary material for: Intravenous methylprednisolone pulse as a treatment for hospitalised severe COVID-19 patients: results from a randomised controlled clinical trial
Source: Eur Respir J. 2020 Dec 24;56(6):2002808. doi: 10.1183/13993003.02808-2020 (PMC7758541; doi:10.1183/13993003.02808-2020)
Supplement: Supplementary file 3 [file ERJ-02808-2020.Figure_S3.pdf]

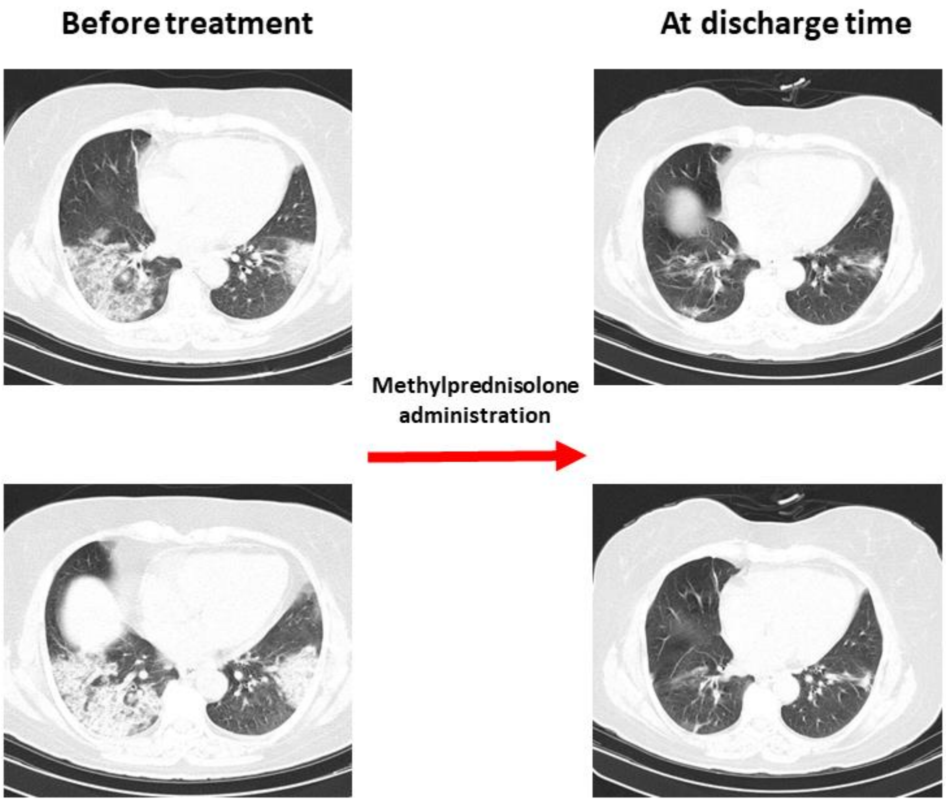

Supplementary Figure 3. Improved pulmonary involvement in a chest computed tomography (CT) of a 55 years old, confirmed COVID-19 patient from methylprednisolone group after 21 days of treatment (at discharge time).
