# Supplementary material for: Intravenous methylprednisolone pulse as a treatment for hospitalised severe COVID-19 patients: results from a randomised controlled clinical trial
Source: Eur Respir J. 2020 Dec 24;56(6):2002808. doi: 10.1183/13993003.02808-2020 (PMC7758541; doi:10.1183/13993003.02808-2020)
Supplement: Supplementary file 4 [file ERJ-02808-2020.Table_S1.pdf]

Supplementary Table 1. Summary of adverse events in patients.

**Characteristic**	**Methylprednisolone (N=34)**	**Standard care (N=28)**	***P* value**
**Infection, no (%)**	1 (2.9%)	0	0.548
**Edema, no (%)**	1(2.9%)	0	0.548
**Shock, no (%)**	0	2 (7.1%)	0.208
**Digestive bleeding, no (%)**	0	0	NA
**Others**	0	0	NA

NA: Not applicable
