# Supplementary material for: Intravenous methylprednisolone pulse as a treatment for hospitalised severe COVID-19 patients: results from a randomised controlled clinical trial
Source: Eur Respir J. 2020 Dec 24;56(6):2002808. doi: 10.1183/13993003.02808-2020 (PMC7758541; doi:10.1183/13993003.02808-2020)
Supplement: Supplementary file 5 [file ERJ-02808-2020.Table_S2.pdf]

Supplementary Table 2. Clinical characteristics of the recovered patients one week after discharge (Methylprednisolone n=32, Standard care n=16).

Characteristic	Before treatment				One week after discharge					
	Methylprednisolone		Standard care		Methylprednisolone		*P* value	Standard care		*P* value
	Mean	SD	Mean	SD	Mean	SD		Mean	SD	
**BORG score**	7.6	1.7	6.43	2.6	0.75	0.62	**<0.001**	1	0.63	**0.001**
	no	%	no	%	no	%		no	%	
**GI Symptom**	16	50	7	43.8	0	0	**0.016**	1	6.3	0.50
**Myalgia**	17	53.1	7	43.8	0	0	**0.043**	1	6.3	0.50
**Chest pain**	7	22	3	18.8	0	0	0.125	0	0	0.69
**Cough**	22	68.8	9	56.3	6	18.8	0.250	3	18.8	0.55

P value in the bold form is statistically significant (*P* value< 0.05)
SD, Standard deviation; GI, Gastrointestinal;
