# Supplementary material for: Intravenous methylprednisolone pulse as a treatment for hospitalised severe COVID-19 patients: results from a randomised controlled clinical trial
Source: Eur Respir J. 2020 Dec 24;56(6):2002808. doi: 10.1183/13993003.02808-2020 (PMC7758541; doi:10.1183/13993003.02808-2020)
Supplement: Supplementary file 6 [file ERJ-02808-2020.Shareable.pdf]

SHAREABLE PDF

Intravenous methylprednisolone pulse as a treatment for hospitalised severe COVID-19 patients: results from a randomised controlled clinical trial

Maryam Edalatifard[1,17], Maryam Akhtari[2,3,17], Mohammadreza Salehi[4], Zohre Naderi[5], Ahmadreza Jamshidi[2,18], Shayan Mostafaei[6], Seyed Reza Najafizadeh[7], Elham Farhadi[2,3], Nooshin Jalili[8], Masoud Esfahani[9], Besharat Rahimi[1], Hossein Kazemzadeh[1], Maedeh Mahmoodi Aliabadi[10], Tooba Ghazanfari[11], Mohammadreza Sattarian[12], Hourvash Ebrahimi Louyeh[13], Seyed Reza Raeeskarami[14], Saeidreza Jamalimoghadamsiahkali[15], Nasim Khajavirad[16], Mahdi Mahmoudi[2,3,18] and Abdolrahman Rostamian[7,18]

Affiliations: [1]Advanced Thoracic Research Center, Tehran University of Medical Sciences, Tehran, Iran. [2]Rheumatology Research Center, Tehran University of Medical Sciences, Tehran, Iran. [3]Inflammation Research Center, Tehran University of Medical Sciences, Tehran, Iran. [4]Dept of Infectious and Tropical Medicines, Tehran University of Medical Sciences, Tehran, Iran. [5]Dept of Internal Medicine, Isfahan University of Medical Sciences, Isfahan, Iran. [6]Dept of Biostatistics, School of Health, Kermanshah University of Medical Sciences, Kermanshah, Iran. [7]Rheumatology Research Center, Imam Khomeini Hospital, Tehran University of Medical Sciences, Tehran, Iran. [8]Dept of Internal Medicine, School of Medicine, Zanjan University of Medical Sciences, Zanjan, Iran. [9]Dept of Clinical Pharmacy, Faculty of Pharmacy, Tehran University of Medical Sciences, Tehran, Iran. [10]Dept of Laboratory, Imam Khomeini Hospital Complex, Tehran University of Medical Sciences, Tehran, Iran. [11]Immunoregulation Research Center, Shahed University, Tehran, Iran. [12]Simorgh Clinical Laboratory, Tehran, Iran. [13]Dept of Rheumatology, Imam Khomeini Hospital Complex, Tehran University of Medical Sciences, Tehran, Iran. [14]Dept of Pediatrics, Tehran University of Medical Sciences, Tehran, Iran. [15]Ziaeian Hospital, Tehran University of Medical Sciences, Tehran, Iran. [16]Dept of Internal Medicine, School of Medicine, Tehran University of Medical Sciences, Tehran, Iran. [17]These two authors contributed equally as first authors. [18]These three authors contributed equally as lead authors and supervised the work.

Correspondence: Abdolrahman Rostamian, Rheumatology Research Center, Imam Khomeini Hospital, PO Box 1418419967, Tehran, Iran. E-mail: arostamian@tums.ac.ir

@ERSpublications
This study showed that methylprednisolone pulse administration at the beginning of the early pulmonary phase of illness decreased the mortality rate and improved pulmonary involvement, oxygen saturation and inflammatory markers in COVID-19 patients https://bit.ly/3hik4JB

Cite this article as: Edalatifard M, Akhtari M, Salehi M, *et al*. Intravenous methylprednisolone pulse as a treatment for hospitalised severe COVID-19 patients: results from a randomised controlled clinical trial. *Eur Respir J* 2020; 56: 2002808 [https://doi.org/10.1183/13993003.02808-2020].

This single-page version can be shared freely online.

ABSTRACT

Introduction: There are no determined treatment agents for severe COVID-19. It is suggested that methylprednisolone, as an immunosuppressive treatment, can reduce the inflammation of the respiratory system in COVID-19 patients.

Methods: We conducted a single-blind, randomised controlled clinical trial involving severe hospitalised patients with confirmed COVID-19 at the early pulmonary phase of the illness in Iran. The patients were randomly allocated in a 1:1 ratio by the block randomisation method to receive standard care with methylprednisolone pulse (intravenous injection, 250 $mg \cdot day^{-1}$ for 3 days) or standard care alone. The

Copyright ©ERS 2020. This version is distributed under the terms of the Creative Commons Attribution Non-Commercial Licence 4.0.

study end-point was the time of clinical improvement or death, whichever came first. Primary and safety analysis was done in the intention-to-treat (ITT) population.

Results: 68 eligible patients underwent randomisation (34 patients in each group) from April 20, 2020 to June 20, 2020. In the standard care group, six patients received corticosteroids by the attending physician before the treatment and were excluded from the overall analysis. The percentage of improved patients was higher in the methylprednisolone group than in the standard care group (94.1% *versus* 57.1%) and the mortality rate was significantly lower in the methylprednisolone group (5.9% *versus* 42.9%; $p<0.001$). We demonstrated that patients in the methylprednisolone group had a significantly increased survival time compared with patients in the standard care group (log-rank test: $p<0.001$; hazard ratio 0.293, 95% CI 0.154–0.556). Two patients (5.8%) in the methylprednisolone group and two patients (7.1%) in the standard care group showed severe adverse events between initiation of treatment and the end of the study.

Conclusions: Our results suggest that methylprednisolone pulse could be an efficient therapeutic agent for hospitalised severe COVID-19 patients at the pulmonary phase.
